# Description of Flavobacterium fructosi sp. nov., Flavobacterium xylosi sp. nov. and Flavobacterium zhouii sp. nov., three new members of the genus Flavobacterium

**DOI:** 10.1099/ijsem.0.006694

**Published:** 2025-02-26

**Authors:** Dou Han, Lei-Lei Yang, Yu-Hua Xin, Qing Liu

**Affiliations:** 1 College of Biological Sciences, China Agricultural University, Beijing 100093, PR China; 2 China General Microbiological Culture Collection Center (CGMCC), Institute of Microbiology, Chinese Academy of Sciences, Beijing 100101, PR China

**Keywords:** *Flavobacterium*, *Flavobacterium fructosi*, *Flavobacterium xylosi*, *Flavobacterium zhouii*

## Abstract

Three novel strains, designated LB3P45^T^, LS2P90^T^ and ZS1P70^T^, were isolated from glaciers located on the Tibetan Plateau, PR China. These strains were Gram-stain-negative, aerobic, rod-shaped and yellow or orange coloured. Phylogenetic analysis based on the 16S rRNA gene and genomic sequences indicated that they belong to the genus *Flavobacterium*. The 16S rRNA gene sequence similarity among the three strains ranged from 97.4 to 98.6%. Strain LB3P45^T^ showed 98.9% and 98.7% similarity to *Flavobacterium urumqiense* CGMCC 1.9230^T^ and *Flavobacterium xinjiangense* JCM 11314^T^, respectively. Strain LS2P90^T^ displayed 99.0% and 98.7% similarity to *F. xinjiangense* JCM 11314^T^ and *F. urumqiense* CGMCC 1.9230^T^. Strain ZS1P70^T^ had the highest sequence similarity with *F. xinjiangense* JCM 11314^T^ (98.0%) and *F. urumqiense* CGMCC 1.9230^T^ (97.8%). The average nucleotide identity and digital DNA–DNA hybridization values between these strains and their closest relatives were lower than 93.0% and 47.9%, respectively. All three strains contained summed feature 3 (comprising C_16:1_ *ω7*c and/or C_16:1_ *ω6*c) as the major fatty acids. Based on phenotypic characteristics, phylogenetic analysis and genotypic data, the three novel species are proposed: *Flavobacterium fructosi* sp. nov. (LB3P45^T^=CGMCC 1.11439^T^=NBRC 114819^T^), *Flavobacterium xylosi* sp. nov. (LS2P90^T^=CGMCC 1.11685^T^=NBRC 114823^T^) and *Flavobacterium zhouii* sp. nov. (ZS1P70^T^=CGMCC 1.24124^T^=NBRC 114829^T^).

The genus *Flavobacterium* was first proposed by Bergey *et al*. with *Flavobacterium aquatile* as the type species [1] and has been emended several times [2,4]. *Flavobacterium* strains are Gram-negative, aerobic, rod-shaped and yellow-pigmented. These bacteria have been isolated from diverse environments, including freshwater [5,6], soil [7], lake sediment [8] and glaciers [9]. The genus *Flavobacterium* is particularly abundant in bacterial populations found on glaciers [10]. In addition to *Flavobacterium*, our survey of bacterial diversity on glaciers also identified other genera, such as *Cryobacterium* [11], *Arthrobacter* [12] and *Mucilaginibacter* [13]. At the time of writing, *Flavobacterium* includes 312 species with validly published names [14]. In this study, we isolated and characterized three novel *Flavobacterium* strains – designated LB3P45^T^, LS2P90^T^ and ZS1P70^T^ – from glacier samples using a polyphasic taxonomic approach.

## Isolation and ecology

Strains LB3P45^T^ and LS2P90^T^ were isolated from ice and meltwater samples, respectively, of the Laigu Glacier (29.3087826 N, 96.8186951 E), while strain ZS1P70^T^ was isolated from a meltwater sample of the Zepu Glacier (30.276556 N, 95.2508392 E) on the Tibetan Plateau. During sample collection in October 2016, sterile gloves were worn, and samples were placed in sterile sampling bags. The samples were transported to the laboratory at low temperature and homogenized with sterile water. Tenfold serial dilutions were prepared, and 200 µl of the bacterial suspension was spread on peptone, yeast extract, and glucose (PYG) [12] plates. The plates were incubated at 14 °C for 30 days. Single colonies were selected and purified. For long-term preservation, the strains were stored in preserved 10% (v/v) aqueous glycerol suspensions in a liquid nitrogen storage tank.

## 16S rRNA Phylogeny

The genomic DNA of the three strains was extracted using the TaKaRa MiniBEST Bacteria Genomic DNA Extraction Kit version 3.0 (TaKaRa, Dalian, China) following the manufacturer’s instructions. The 16S rRNA gene was amplified and sequenced using the universal primers 27F and 1492R [15]. The resulting 16S rRNA gene sequences were compared and identified through the EzBioCloud database [16]. Multiple sequence alignments were performed using MAFFT software version 7.520 with default parameters [17]. Phylogenetic trees were generated using neighbour-joining (NJ), maximum likelihood (ML), and maximum parsimony (MP) methods in mega7 [18], with 1000 bootstrap replicates to evaluate the robustness of the tree topology. Kimura’s two-parameter model was employed to calculate genetic distances for the NJ analysis.

**Fig. 1.**
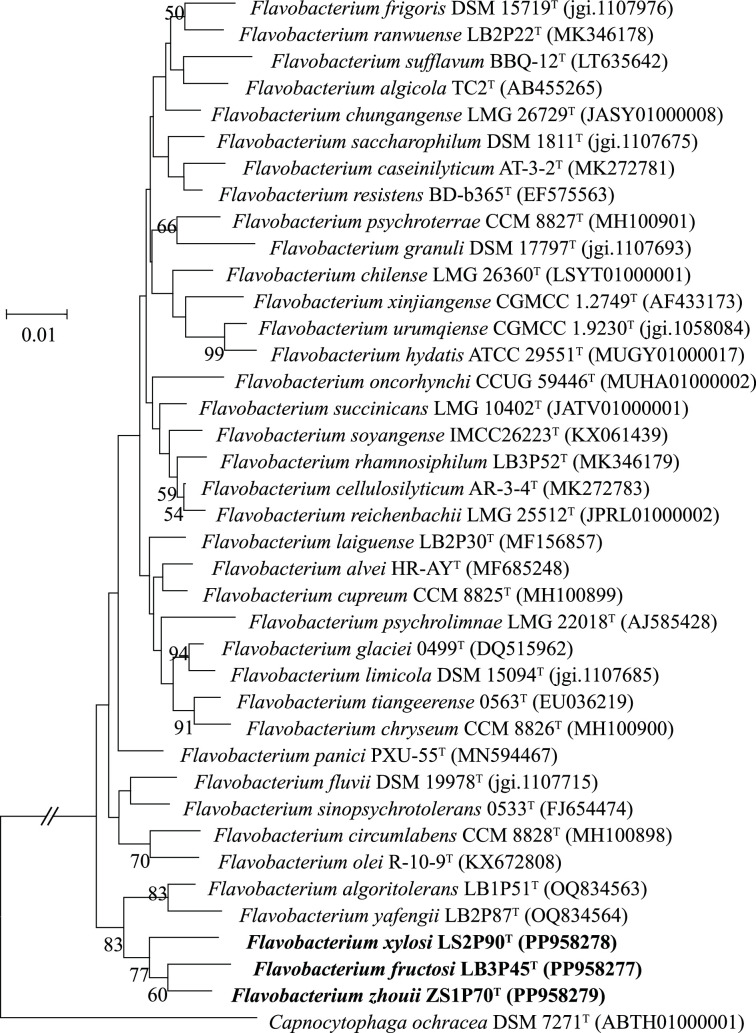
Phylogenetic tree of strains LB3P45^T^, LS2P90^T^ and ZS1P70^T^ and related strains based on 16S rRNA gene sequence comparisons using the NJ method. GenBank accession numbers of the 16S rRNA gene sequences are shown in parentheses. Bootstrap values (>50%) based on 1000 replicates are indicated at the branch nodes. Bar, 0.01 substitutions per nucleotide position.

The almost full-length 16S rRNA gene sequences of LB3P45^T^ (1327 bp), LS2P90^T^ (1375 bp) and ZS1P70^T^ (1357 bp) were acquired and compared with the EzBioCloud database. The 16S rRNA gene sequence comparisons indicated that they are classified within the genus *Flavobacterium*. The 16S rRNA gene sequences exhibited a similarity of 97.4–98.6% among the three strains, suggesting they likely belong to different species. Strain LB3P45^T^ showed the closest relation to *Flavobacterium urumqiense* CGMCC 1.9230^T^ (98.9%), *Flavobacterium xinjiangense* JCM 11314^T^ (98.7%) and *Flavobacterium sinopsychrotolerans* 0533^T^ (97.8%). Strain LS2P90^T^ was closest to *F. xinjiangense* JCM 11314^T^ (99.1%), *F. urumqiense* CGMCC 1.9230^T^ (98.7%) and *F. sinopsychrotolerans* 0533^T^ (98.3%). Strain ZS1P70^T^ exhibited the highest sequence similarity with *F. xinjiangense* JCM 11314^T^ (98.0%), followed by * F. urumqiense* CGMCC 1.9230^T^ (97.8%) and *Flavobacterium psychrolimnae* LMG 22018^T^ (97.3%). Strains LS2P90^T^, LB3P45^T^ and ZS1P70^T^ clustered together in the NJ tree (Fig. 1), as well as in the ML (Fig. S1, available in the online Supplementary Material) and MP trees (Fig. S2). These three strains formed a bigger clade with *Flavobacterium algoritolerans* LB1P51^T^ and *Flavobacterium yafengii* LB2P87^T^ in both the NJ and ML trees, but not in the MP tree.

## Genome features

To assess the genetic relationships between the three strains and their relatives, genome sequencing was performed using the Illumina HiSeq 4000 platform (Illumina, San Diego, CA, USA) following the manufacturer’s protocols for 150 bp paired-end reads. Short reads were *de novo* assembled using the SPAdes program v3.15 [19]. The CheckM2 v1.0.2 program [20] was used to check the completeness and contamination values of the genomes. The quality of the assemblies was assessed using QUAST v5.0.2 [21]. A phylogenomic tree was constructed using IQ-TREE 2 software [22] based on the concatenated sequence dataset of 81 single-copy core genes extracted by the UBCG2 pipeline [23]. The alignments were generated using MAFFT v7.520 software [17]. The tree was evaluated by 1000 bootstrap replicates with the best model TIM2+F+R6. The average nucleotide identity (ANI) value was calculated using the FastANI program [24]. The digital DNA–DNA hybridization (dDDH) value was calculated using the Type (Strain) Genome Server [25]. Gene prediction and annotation were performed using the Prokka v1.14 software [26]. The genome was also annotated with the Kyoto Encyclopedia of Genes and Genomes (KEGG) pathway using eggNOG-mapper v2.1.9 [27].

**Fig. 2.**
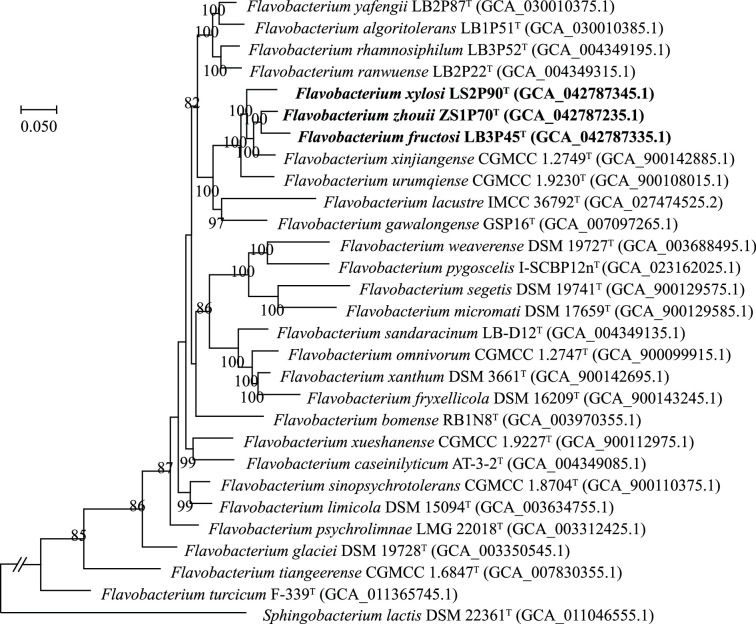
Phylogeny of strains LB3P45^T^, LS2P90^T^ and ZS1P70^T^ and related species inferred using the ML method with the best-fitting model TIM2+F+R6, based on the concatenated alignment of 81 core genes. Bootstrap values (>70%) based on 1000 replicates are shown at the branch nodes. Bar, 0.05 substitutions per position.

The completeness of the genome sequence for the three strains was 100%, with contamination levels ranging from 0.87 to 1.13%. The assembled genome information of the three strains is provided in Table S1. Their G+C content is 34 mol%. They have similar genome sizes, ranging from 3.65 to 3.69 Mb, consisting of 55 to 114 contigs. Each strain has over 3200 annotated protein-coding sequences. A robust phylogenomic tree was reconstructed based on the concatenated 81 core genes, revealing that the three strains and their closest relatives, *F. xinjiangense* CGMCC 1.2749^T^ and *F. urumqiense* CGMCC 1.9230^T^, formed a small clade (Fig. 2). They clustered together with six other species with validly published names, creating a distinct lineage with a bootstrap value of 82%. Notably, all type strains in this lineage, except for *Flavobacterium lacustre* IMCC 36792^T^, were isolated from glacial environments. This suggested that species originating from the cryosphere may have formed ecological niches that reflect their phylogenetic lineage.

The pairwise ANI and dDDH values between strains LS2P90^T^, LB3P45^T^ and ZS1P70^T^ and their closest relatives, *F. algoritolerans* LB1P51^T^, *F. yafengii* LB2P87^T^, *F. xinjiangense* CGMCC 1.2749 ^T^ and *F. urumqiense* CGMCC 1.9230^T^, are presented in Table 1. These values meet the established criteria for classifying a strain as a distinct species, specifically an ANI value of less than 95% and a dDDH value of less than 70% [28,29]. This result indicated that strains LB3P45^T^, LS2P90^T^ and ZS1P70^T^ represent three novel species within the genus *Flavobacterium*.

**Table 1.** The pairwise ANI (lower left ‘triangle’, %) and dDDH (upper right ‘triangle’, %) values between the three strains and their closest relatives Strains: 1, LB3P45^T^; 2, LS2P90^T^; 3, ZS1P70^T^; 4, *F. urumqiense* CGMCC 1.9230^T^; 5, *F. xinjiangense* CGMCC 1.2749^T^; 6, *F. algoritolerans* LB1P51^T^; 7, *F. yafengii* LB2P87^T^.

	1	2	3	4	5	6	7
**1**		33.2	46.3	36.3	39.6	25.0	25.2
**2**	87.7		34.9	34.6	35.1	25.8	25.6
**3**	92.6	88.6		35.1	47.9	25.1	25.2
**4**	89.3	88.1	88.5		33.9	25.1	25.4
**5**	90.4	88.7	93.0	87.9		24.9	24.9
**6**	82.9	83.4	82.7	82.8	82.9		45.6
**7**	82.7	83.2	82.8	83.0	83.0	92.1	

The genomes of the three strains, LB3P45^T^, LS2P90^T^ and ZS1P70^T^, were annotated for metabolic pathways using the KEGG database (Fig. S3). Strain LB3P45^T^ exhibited 262 genes related to carbohydrate metabolism and 228 for amino acid metabolism, while LS2P90^T^ had 241 and 228 genes, respectively. ZS1P70^T^ contained 259 genes for carbohydrate metabolism and 223 for amino acid metabolism. In terms of energy metabolism, LB3P45^T^ had 132 genes, LS2P90^T^ had 129 and ZS1P70^T^ had 133. For cofactor and vitamin metabolism, the counts were 128, 118 and 123 genes, respectively. Each strain also contained pathways for lipid, nucleotide and other amino acid metabolisms, with slight variations in gene counts. Regarding environmental adaptation, LB3P45^T^ and LS2P90^T^ each had 37 and 35 genes for membrane transport, while ZS1P70^T^ had 40. Signal transduction genes numbered 48 in LB3P45^T^, 52 in LS2P90^T^ and 51 in ZS1P70^T^. Notably, for xenobiotic biodegradation and metabolism, LB3P45^T^ had 37 genes, LS2P90^T^ had 40 and ZS1P70^T^ had 34, suggesting potential bioremediation capabilities. Additionally, terpenoid metabolism was represented by 28 genes in LB3P45^T^, 26 in LS2P90^T^ and 25 in ZS1P70^T^.

## Physiology and chemotaxonomy

Strains *F. algoritolerans* LB1P51^T^ (=CGMCC 1.11237^T^=NBRC 114813^T^) and *F. yafengii* LB2P87^T^ (=CGMCC 1.11249^T^=NBRC 114814^T^) [30], which were obtained from the China General Microbiological Culture Collection Center, were used as reference strains for phenotypic tests. The colony morphological characteristics of the three novel strains were observed on PYG agar plates. Gram staining was conducted following standard procedures [31]. Cell morphology was observed using a JEM-1400 transmission electron microscope (JEOL Ltd., Tokyo, Japan) in transmission electron microscopy, as shown in Fig. S4. Gliding motility was observed using oil-immersion phase-contrast microscopy after incubation on 1/4 Reasoner’s 2A agar following the protocol by Bernardet *et al.* [32]. Cell growth in PYG broth was assessed at different temperatures (0, 4, 14, 18, 19, 20, 21, 23 and 25 °C). The growth pH range was evaluated in PYG broth adjusted to pH 4.0–10.0 (at 1.0 pH unit intervals). Appropriate biological buffers (0.2 M Na_2_HPO_4_/NaH_2_PO_4_ for pH 5–8 and 0.2 M Na2CO3/NaHCO3 for pH 9–10) were used to adjust the PYG broth. The NaCl tolerance range (0–4% w/v, in 0.5% intervals) was assessed in the PYG medium. Flexirubin-type pigment was detected using 20% KOH (w/v) [31]. The enzymatic activities for starch, casein and Tween 80 hydrolysis were evaluated as described previously [31]. Cytochrome oxidase activity was assessed with 1% (w/v) tetramethyl-p-phenylenediamine (bioMérieux). Catalase activity was determined by observing bubble production in 3% (v/v) H_2_O_2_. The utilization of a single carbon source was tested using a basal medium (containing 0.2% (NH_4_)2SO_4_, 0.05% NaH_2_PO_4_·H_2_O, 0.05% K_2_HPO_4_, 0.02% MgSO_4_·7H_2_O and 0.01% CaCl_2_·2H_2_O) with 1% (w/v) of each carbon compound. The API 20NE, API 20E and API ZYM strips (bioMérieux, Marcy l'Étoile, France) were utilized for additional biochemical tests following the manufacturer’s instructions. For cellular fatty acid analysis, cell masses of the three novel strains and the reference strain were harvested during the late exponential growth phase in PYG medium at 14 °C. Cellular fatty acids were extracted and analysed following the protocol outlined by the MIDI 6.0 system (MIDI Inc., Newark, DE, USA) [33]. The samples were subjected to gas chromatography using an Agilent 6890 N Gas Chromatograph (Agilent Technologies, Santa Clara, CA, USA) with the TSBA6 database for analysis.

Strains LB3P45^T^, LS2P90^T^ and ZS1P70^T^ were Gram-stain-negative, aerobic and rod-shaped bacteria without flagella. Flexirubin-type pigments were absent. Strain LB3P45^T^ could grow at temperatures ranging from 0 to 21 °C, strain LS2P90^T^ could grow from 0 to 19 °C, while strain ZS1P70^T^ could grow from 0 to 23 °C. Specific phenotypic characteristics of the three new strains are outlined in the species description. Strains LB3P45^T^, LS2P90^T^ and ZS1P70^T^ and the reference strain could be differentiated from each other as shown in Table 2. The major cellular fatty acids (>10%) of strain LB3P45^T^ included summed feature 3 (31.4%; comprising C_16:1_ *ω7*c and/or C_16:1_ *ω6*c), *iso*-C_15:0_ (13.1%) and *iso*-C_15:0_ 3-OH (10.5%). Strain LS2P90^T^ displayed the predominant cellular fatty acids (>10%) such as summed feature 3 (23.8%; comprising C_16:1_ *ω7*c and/or C_16:1_ *ω6*c) and *anteiso*-C_15:0_ (19.2%). The predominant cellular fatty acids (>10%) of strain ZS1P70^T^ were summed feature 3 (15.8%; comprising C_16:1_ *ω7*c and/or C_16:1_ *ω6*c), *iso*-C_15:0_ (12.3%) and C_17:1_ *ω6*c (10.7%). The fatty acid profiles show variability among the three strains and reference strains. Summed feature 3 was significantly present in all strains, with the highest concentration found in strain LB3P45^T^ (31.4%). *Anteiso*-C_15:0_ exhibited notable variation, with strain LS2P90^T^ having the highest amount (19.2%), while the other four strains display lower levels ranging from 5.3 to 7.1%. The detailed cellular fatty acid compositions of the five strains are presented in Table S2.

**Table 2.** Differences in phenotypic characteristics among the three novel strains and the reference strains 1, LB3P45^T^; 2, LS2P90^T^; 3, ZS1P70^T^; 4, *F. algoritolerans* LB1P51^T^; 5, *F. yafengii* LB2P87^T^. +, Positive; –, negative.

Characteristic	1	2	3	4	5
Gliding motility	+	−	−	−	−
Cell size (μm)	(0.8–0.9)×(2.1–4.9)	(0.9–1.2)×(1.4–2.1)	(0.8–0.9)×(2.2–3.1)	0.7×(2.3–3.0)	(0.4–0.5)×(3.1–3.7)
Growth temperature range (℃)	0–21	0–19	0–23	0–25	0–27
pH range for growth	5.0–8.0	5.0–7.0	5.0–8.0	6.0–8.0	6.0–9.0
NaCl range for growth (w/v, %)	0–0.5	0–0.05	0–0.5	0–1.0	0–2.0
Voges–Proskauer test	+	+	−	+	+
Reduction of nitrates to nitrites	+	−	+	−	−
Hydrolysis of gelatin	+	+	−	+	+
Acid produced from d-glucose	−	−	−	+	−
**Enzyme activity:**					
Esterase (C4)	−	−	−	+	+
Esterase lipase (C8)	−	−	−	+	+
Lipase (C14)	−	−	−	+	+
Trypsin	−	−	−	+	+
* α*-Chymotrypsin	+	+	−	−	+
* β*-Galactosidase	+	−	+	+	+
* β*-Glucosidase	−	−	+	+	+
**Utilization of:**					
d-Xylose	−	+	−	−	−
d-Turanose	+	+	−	+	+
d-Fructose	+	+	−	+	+
l-Rhamnose	−	+	−	−	+
l-Arabinose	+	+	−	+	+

Based on phylogenetic, physiological, chemotaxonomic and genotypic characteristics, strains LB3P45^T^, LS2P90^T^ and ZS1P70^T^ were identified as three novel species within the genus *Flavobacterium*. The names *Flavobacterium fructosi* sp. nov. (type strain=LB3P45^T^), *Flavobacterium xylosi* sp. nov. (type strain=LS2P90^T^) and *Flavobacterium zhouii* sp. nov. (type strain=ZS1P70^T^) are proposed for the novel species, respectively.

## Protologue

### Description of *Flavobacterium fructosi* sp. nov.

*Flavobacterium fructosi* (fruc.to’si. N.L. gen. n. *fructosi*, of fructose).

Cells are Gram-stain-negative, aerobic and rod-shaped and exhibit gliding motility devoid of flagella, measuring 0.8–0.9 µm×2.1–4.9 µm. Colonies are yellow, convex, round and 3.0 mm in diameter after 7-day incubation on PYG plates at 14 °C. Growth occurs at temperatures between 0 and 21 °C (optimum 14 °C), at pH 5.0–8.0 (optimum pH 7.0) and in the presence of 0–0.5 % (w/v) NaCl. Flexirubin-type pigments are absent. Positive for oxidase and catalase. Does not hydrolyse starch, casein or Tween 80. No acid production is observed from amygdalin, d-mannitol, d-melibiose, d-sorbitol, d-sucrose, glucose, inositol, l-arabinose and l-rhamnose. Indole and H_2_S are not formed. Positive for alkaline phosphatase, leucine arylamidase, valine arylamidase, cystine arylamidase, *α*-chymotrypsin, acid phosphatase, naphthol-AS-BI-phosphohydrolase, *α*-glucosidase, *N*-acetyl-*β*-glucosaminidase, *β*-galactosidase, Voges–Proskauer test, hydrolysis of gelatin, reduction of nitrates to nitrites and hydrolysis of aesculin. Utilize the following carbon sources: d-glucose, d-galactose, melibiose, maltose, lactose, d-mannose, d-turanose, d-fructose, glycogen, l-proline, d-raffinose, sucrose, d-trehalose, cellobiose and l-arabinose. The major fatty acids (>5%) are summed feature 3 (comprising C_16:1_ *ω7*c and/or C_16:1_ *ω6*c), *iso*-C_15:0_, *iso*-C_15:0_ 3-OH, *anteiso*-C_15:0_, C_15:1_ *ω6*c and C_17:1_ *ω6*c. The DNA G+C content of the type strain is 34 mol%.

The type strain LB3P45^T^ (=CGMCC 1.11439^T^=NBRC 114819^T^) was isolated from an ice sample collected from the Laigu glacier on the Tibetan Plateau, PR China. The NCBI accession numbers for the 16S rRNA gene and genome sequences are PP958277 and JBHZQA000000000, respectively.

### Description of *Flavobacterium xylosi* sp. nov.

*Flavobacterium xylosi* (xy.lo’si. N.L. gen. n. *xylosi*, of xylose).

Cells are Gram-stain-negative, aerobic, rod-shaped, devoid of flagella and non-motile, measuring 0.9–1.2 µm×1.4–2.1 µm. Colonies are orange, convex, round and 2.0 mm in diameter after 7-day incubation on PYG plates at 14 °C. Growth occurs at temperatures between 0 and 19 °C (optimum 14 °C), at pH 5.0–7.0 (optimum pH 7.0) and in the presence of 0–0.0.5% (w/v) NaCl. Flexirubin-type pigments are absent. Positive for oxidase and catalase. Does not hydrolyse starch, casein or Tween 80. No acid production is observed from amygdalin, d-mannitol, d-melibiose, d-sorbitol, d-sucrose, glucose, inositol, l-arabinose and l-rhamnose. Indole and H_2_S are not formed. Positive for alkaline phosphatase, leucine arylamidase, valine arylamidase, cystine arylamidase, *α*-chymotrypsin, acid phosphatase, naphthol-AS-BI-phosphohydrolase, *α*-glucosidase, *N*-acetyl-*β*-glucosaminidase, Voges–Proskauer test, hydrolysis of aesculin and hydrolysis of gelatin. Utilize the following carbon sources: d-glucose, d-galactose, melibiose, maltose, lactose, d-mannose, d-xylose, d-turanose, d-fructose, glycogen, l-rhamnose, l-proline, d-raffinose, sucrose, d-trehalose, cellobiose and l-arabinose. The major fatty acids (>5%) are summed feature 3 (comprising C_16:1_ *ω7*c and/or C_16:1_ *ω6*c), *anteiso*-C_15:0_, *iso*-C_15:0_, *iso*-C_15:0_ 3-OH and *iso*-C_17:0_ 3-OH. The DNA G+C content of the type strain is 34 mol%.

The type strain LS2P90^T^ (=CGMCC 1.11685^T^=NBRC 114823^T^) was isolated from a meltwater sample collected from the Laigu glacier on the Tibetan Plateau, PR China. The NCBI accession numbers for the 16S rRNA gene and genome sequences are PP958278 and JBHZPZ000000000, respectively.

### Description of *Flavobacterium zhouii* sp. nov.

*Flavobacterium zhouii* (zhou’i.i. N.L. gen. n. *zhouii* of Zhou, named in honour of Professor Pei-Jin Zhou, for his contributions to the study of extremophiles).

Cells are Gram-stain-negative, aerobic, rod-shaped, devoid of flagella and non-motile, measuring 0.8–0.9 µm×2.2–3.1 µm. Colonies are yellow, convex, round and ~2.0–3.0 mm in diameter after 7-day incubation on PYG plates at 14 °C. Growth occurs at temperatures between 0 and 23 °C (optimum 14 °C), at pH 5.0–8.0 (optimum pH 7.0) and in the presence of 0–0.5% (w/v) NaCl. Flexirubin-type pigments are absent. Positive for oxidase and catalase. Does not hydrolyse starch, casein or Tween 80. No acid production is observed from amygdalin, d-mannitol, d-melibiose, d-sorbitol, d-sucrose, glucose, inositol, l-arabinose and l-rhamnose. Indole and H_2_S are not formed. Positive for alkaline phosphatase, leucine arylamidase, valine arylamidase, cystine arylamidase, acid phosphatase, naphthol-AS-BI-phosphohydrolase, *β*-galactosidase, *α*-glucosidase, *β*-glucosidase, *N*-acetyl-*β*-glucosaminidase, hydrolysis of gelatin, reduction of nitrates to nitrites and hydrolysis of aesculin. Utilize the following carbon sources: d-glucose, d-galactose, melibiose, maltose, lactose, d-mannose, glycogen, l-proline, d-raffinose, sucrose, d-trehalose and cellobiose. The major fatty acids (>5%) are summed feature 3 (comprising C_16:1_ *ω7*c and/or C_16:1_ *ω6*c), *iso*-C_15:0_, C_17:1_ *ω6*c, C_15:1_ *ω6*c, *iso*-C_15:0_ 3-OH, *iso*-C_17:0_ 3-OH, *anteiso*-C_15:0_ and *iso*-C_15:1_ G. The DNA G+C content of the type strain is 34 mol%.

The type strain ZS1P70^T^ (=CGMCC 1.24124^T^=NBRC 114829^T^) was isolated from a melt water sample collected from the Zepu glacier on the Tibetan Plateau, PR China. The NCBI accession numbers for the 16S rRNA gene and genome sequences are PP958279 and JBHZPY000000000, respectively.

## supplementary material

10.1099/ijsem.0.006694 Uncited Supplementary Material 1.

## Abbreviations

ANI: average nucleotide identity
dDDH: digital DNA–DNA hybridization
KEGG: Kyoto Encyclopedia of Genes and Genomes
ML: maximum likelihood
MP: maximum parsimony
NJ: neighbour-joining
PYG: peptone, yeast extract and glucose
