# Supplementary material for: Description of Flavobacterium fructosi sp. nov., Flavobacterium xylosi sp. nov. and Flavobacterium zhouii sp. nov., three new members of the genus Flavobacterium
Source: Int J Syst Evol Microbiol. 2025 Feb 26;75(2):006694. doi: 10.1099/ijsem.0.006694 (PMC11865497; doi:10.1099/ijsem.0.006694)
Supplement: Uncited Supplementary Material 1. [file ijsem-75-06694-s001.pdf]

Description of *Flavobacterium fructosi* sp. nov., *Flavobacterium xylosi* sp. nov., and *Flavobacterium zhouii* sp. nov., three new members of th e genus *Flavobacterium*

Dou Han, Lei-Lei Yang, Yu-Hua Xin* and Qing Liu*

***Corresponding author:**
Qing Liu, Email: liuqing@im.ac.cn
Yu-Hua Xin, Email: xinyh@im.ac.cn

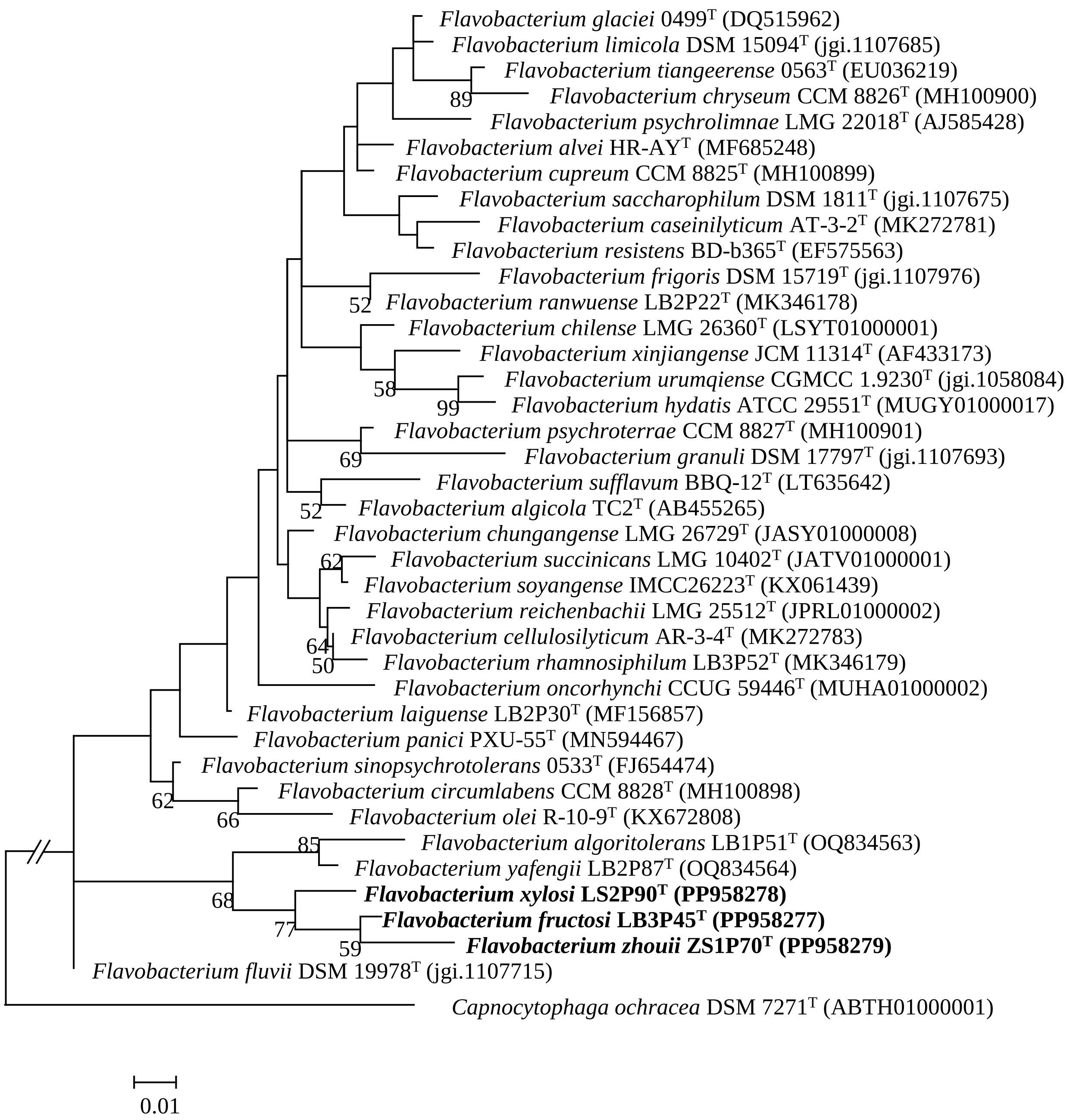

Fig. S1. Phylogenetic tree of the three strains and related strains based on the 16S rRNA gene sequence comparisons using the ML method. GenBank accession numbers of the 16S rRNA gene sequences are given in parentheses. Bootstrap values (>50 %) based on 1,000 replicates are shown at the branch nodes. Bar, 0.01 substitutions per nucleotide positions.

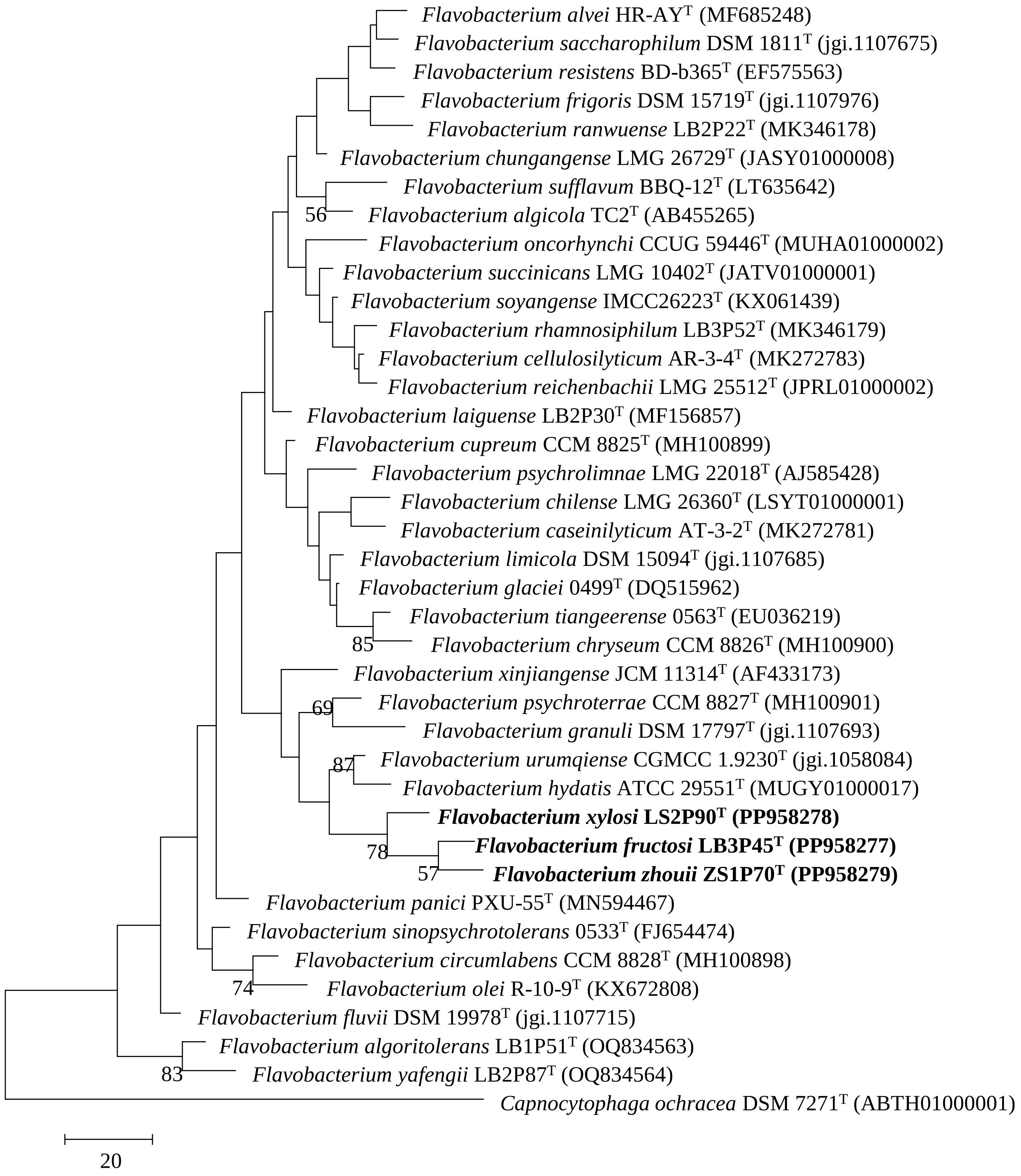

Fig. S2. Phylogenetic tree of the three strains and related strains based on the 16S rRNA gene sequence comparisons using the MP method. GenBank accession numbers of the 16S rRNA gene sequences are given in parentheses. Bootstrap values (>50 %) based on 1,000 replicates are shown at the branch nodes. The tree is drawn to scale , with branch lengths calculated using the average pathway and are in the units of the number of changes over the whole sequence.

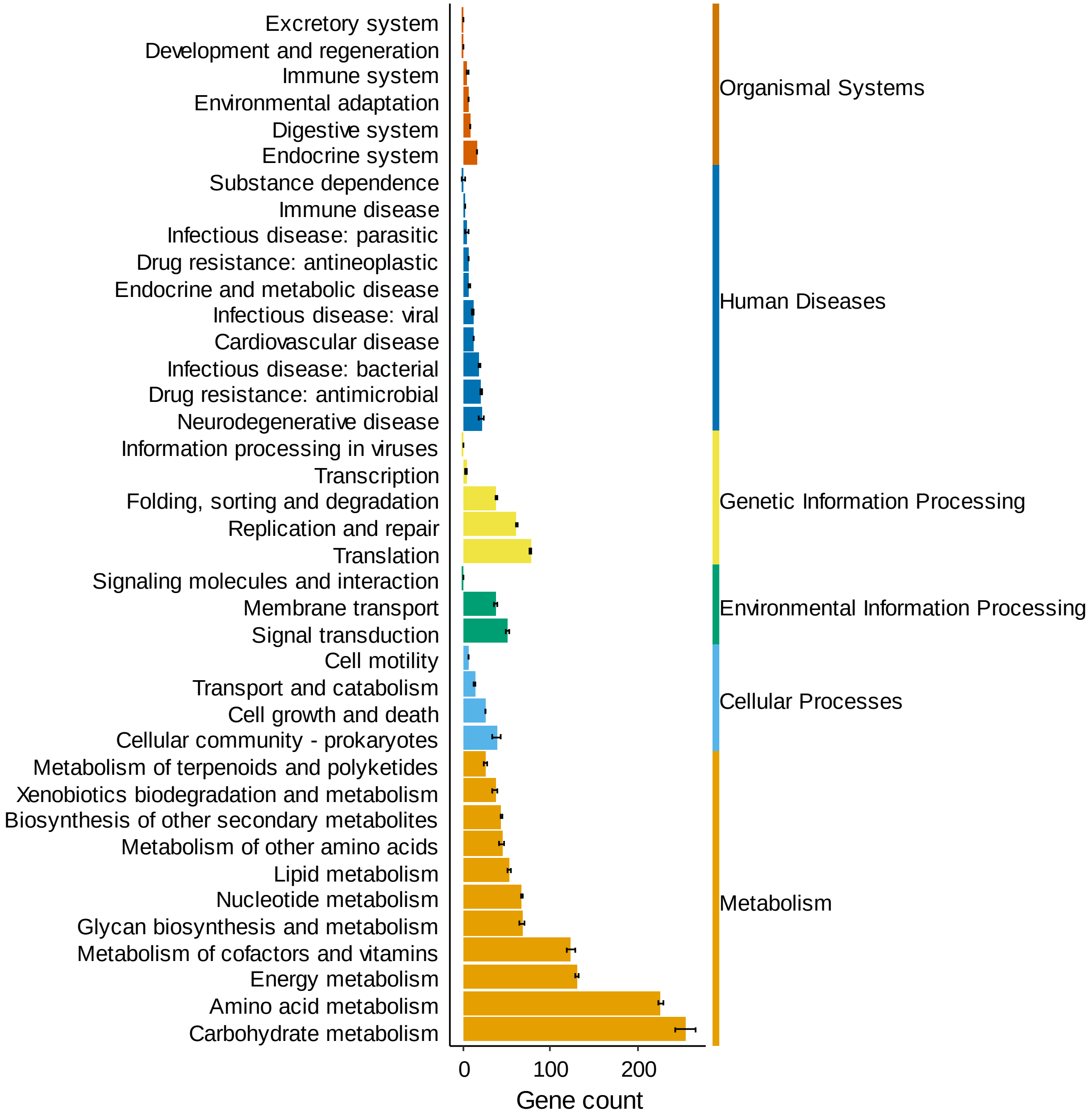

Fig. S3. Metabolic pathway annotations for the twelve strains based on KEGG database. The numbers represent the average gene counts in each category for all strains, with error bars indicating the range among the strains.

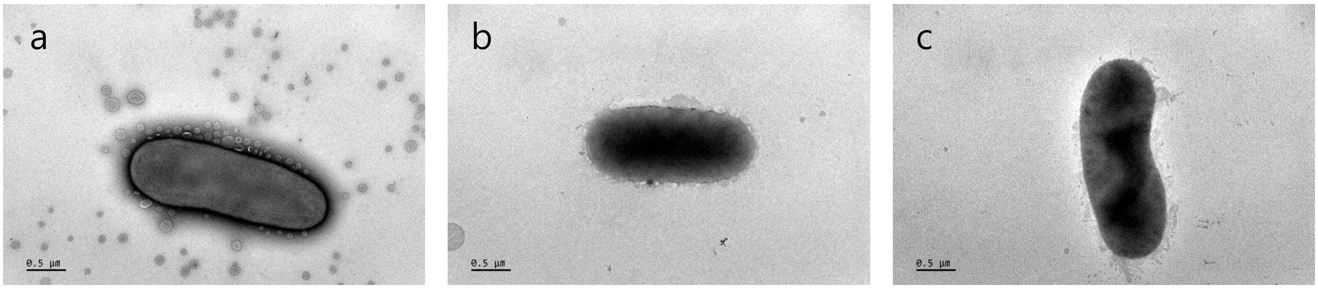

Fig. S4. Transmission electron micrograph of negatively stained cells of strains LB3P45^{T}(a), LS2P90^{T} (b), and ZS1P70^{T} (c) grown at 14℃ on PYG agar. Bar = 0.5 μm.

Table S1. The genome information of strains LB3P45^{T}, LS2P90^{T} and ZS1P70^{T}.

	LB3P45^{T}	LS2P90^{T}	ZS1P70^{T}
**Size (Mb)**	3.65	3.69	3.68
**GC (%)**	34.0	34.2	34.0
**No. of contigs**	60	114	55
**N50 (bp)**	309,918	167,277	254,912
**No. of**			
protein-coding genes	3,249	3,275	3,242
tRNA	45	43	45
5S rRNA	2	3	3
16S rRNA	1	1	1
23S rRNA	1	1	1
tmRNA	-	1	1

Table S2. Cellular fatty acid composition of the three strains and the reference strain.

Strains: 1, LB3P45^T; 2, LS2P90^T; 3, ZS1P70^T; 4, *Flavobacterium algoritolerans* LB1P51^T; 5, *Fla vobacterium yafengii* LB2P87^T. Major fatty acids (>10 %) are represented in bold. TR, traces (<1 %). –, Not detected.

*Summed Features are fatty acids that cannot be resolved reliably from another fatty acid using the chromatographic conditions chosen. The MIDI system groups these fatty acids together as one feature with a single percentage of the total. Summed features consist of: 2, *iso*-$C_{16:1}$ I/ $C_{14:0}$ 3-OH; 3, $C_{16:1}$ $\omega7c$/$C_{16:1}$ $\omega6c$; 4, $C_{17:1}$ iso I/anteiso B/ $C_{17:1}$ anteiso B/iso I; 9, iso-$C_{17:1}$ $\omega9c$/10-methyl $C_{16:0}$.

Fatty acid	1	2	3	4	5
**Saturated**					
$C_{14:0}$	1.4	Tr	Tr	Tr	Tr
$C_{16:0}$	2.3	1.4	1.9	1.7	1.9
**Branched**					
iso-$C_{14:0}$	1.1	1.1	1.1	4.8	1.1
iso-$C_{15:0}$	**13.1**	9.8	**12.3**	**16**	**12.6**
anteiso-$C_{15:0}$	7.1	**19.2**	5.3	5.3	5.6
iso-$C_{16:0}$	Tr	1.2	2.1	1.5	2
**Unsaturated**					
$C_{13:1}$ at 12-13	1.4	Tr	Tr	-	-
iso-$C_{15:1}$ G	3.5	3.6	5.5	8.0	6.0
anteiso-$C_{15:1}$ A	Tr	1.9	Tr	Tr	Tr
iso-$C_{16:1}$ H	-	1.5	1.5	1.2	2.6
$C_{15:1}$ ω6c	7.8	1.9	7.2	3.6	4.6
$C_{17:1}$ ω6c	6.5	2.7	**10.7**	5.5	8.7
$C_{17:1}$ ω8c	Tr	Tr	2.7	-	-
anteiso-$C_{17:1}$ ω9c	-	1.0	-	-	-
**Hydroxy**					
$C_{15:0}$ 2-OH	1.2	1.2	Tr	Tr	Tr
$C_{15:0}$ 3-OH	2.1	-	1.7	-	-
iso-$C_{15:0}$ 3-OH	**10.5**	6.6	7.3	**10.8**	7.4
$C_{16:0}$ 3-OH	-	1.7	1.6	1.4	1.3
$C_{17:0}$ 2-OH	-	1.6	Tr	Tr	Tr
iso-$C_{17:0}$ 3-OH	-	5.8	7.9	6.2	7.1
iso-$C_{16:0}$ 3-OH	2.1	4.9	4.3	4	4.3
**Summed feature ***					
2	2.6	Tr	Tr	-	-
3	**31.4**	**23.8**	**15.8**	**11.0**	**16.7**
4	-	Tr	-	1.7	2.1
9	1.4	3.7	4.3	4.8	7.9
